# Supplementary material for: Breast Cancer Cell Type and Biomechanical Properties of Decellularized Mouse Organs Drives Tumor Cell Colonization
Source: Cells. 2023 Aug 9;12(16):2030. doi: 10.3390/cells12162030 (PMC10453279; doi:10.3390/cells12162030)
Supplement: Supplementary file 1 [file cells-12-02030-s001.zip › cells-2516188-supplementary.pdf]

Supplementary Information for

Breast Cancer Cell Type and Biomechanical Properties of Decellularized Mouse Organs Drives Tumor Cell Colonization

Anton D. Pospelov, Olga M. Kutova, Yuri M. Efremov, Albina A. Nekrasova, Daria B. Trushina, Sofia D. Gefter, Elena I. Cherkasova, Lidia B. Timofeeva, Peter S. Timashev, Andrei V. Zvyagin, Irina V. Balalaeva

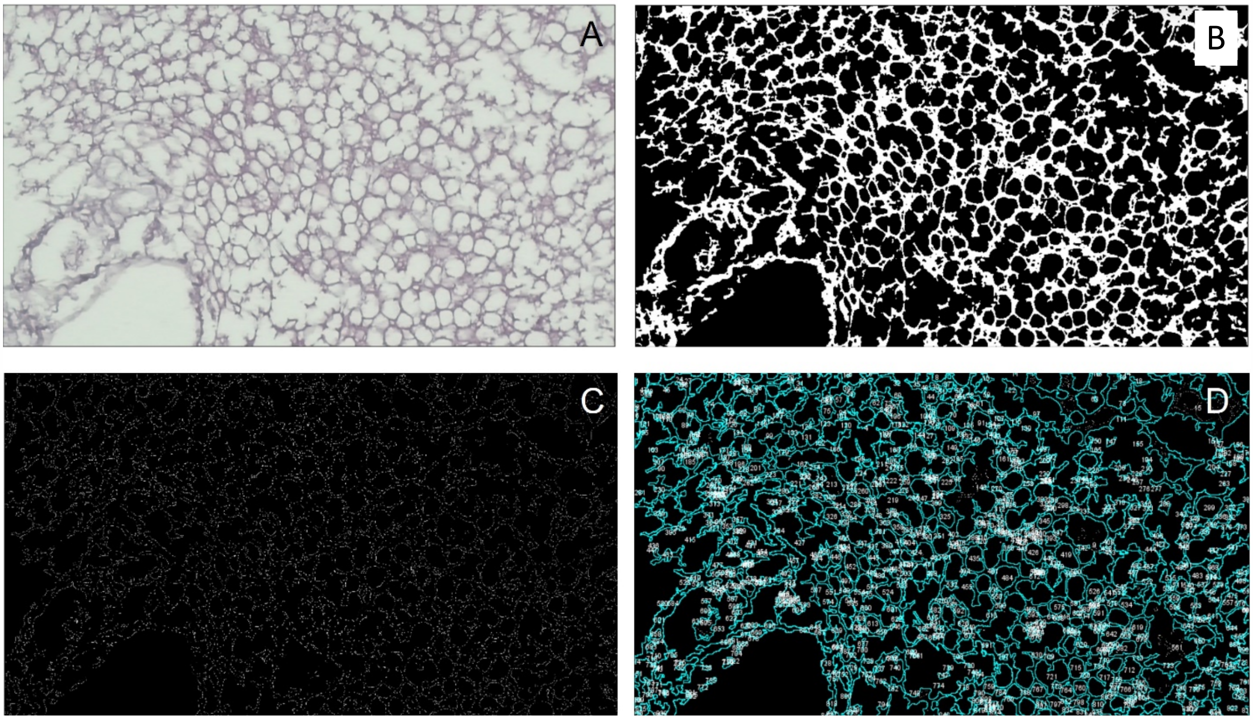

Figure S1. Step-by-step strategy for analyzing images of the DCL matrix in the ImageJ program. The initial histological image of the matrix (A) was converted to 8 bits image, inverted, and changed to black and white with adjusted threshold to ensure the visual integrity of the pores and low back-ground noise (Process-Binary-Make binary; Image-Adjust-Threshold) (B). Each pore and fiber was outlined to make sure, that the program will be able to calculate the area (Process-Binary-Outline) (C). The area of the individual pores was calculated automatically with the set of following settings: Include holes and Show overlay. These two are needed for the program measure each hole and at the same time we can check if the program has chosen right objects as a holes (Analyze-Analyze particles–Show overlay+Include holes) (D).

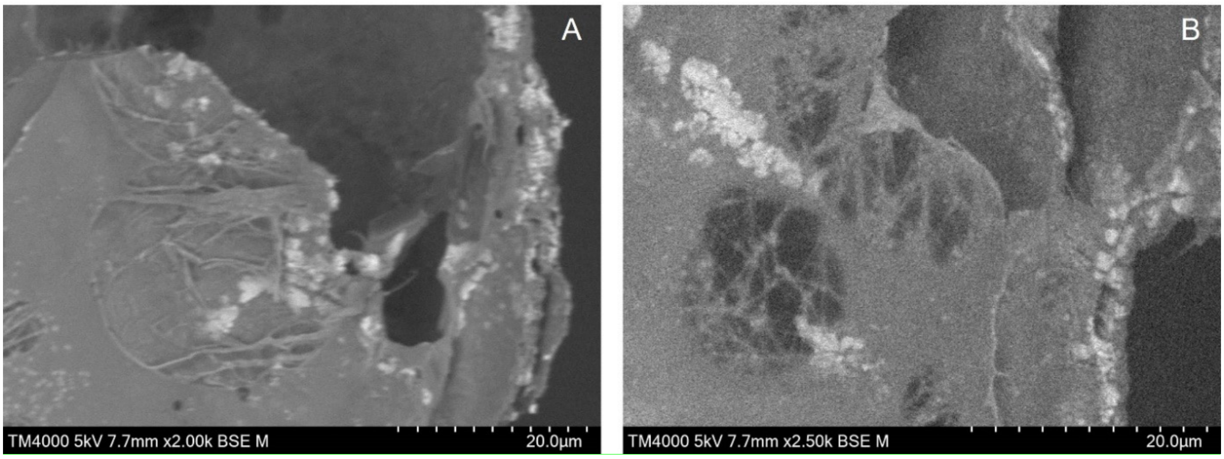

Figuere S2. SEM analysis of a collagen matrix. Images were obtained with magnification ×2000 (A) and ×2500 (B).

	Lung	Spleen	Kidney	Ovary
Liver	*	—	—	*
Ovary	*	*	*	
Kidney	*	—		
Spleen	*			

Figure S3. Results of statistical comparison between the averaged pore areas measured by SEM for matrices of different organs. *, a significant difference between matrices, $p < 0.05$; —, no significant difference is found (one-way ANOVA with Holm-Sidak test for multiple comparisons)

	Lung	Spleen	Kidney	Ovary
Liver	*	*	*	*
Ovary	*	*	*	
Kidney	*	*		
Spleen	*			

Figure S4. Results of statistical comparison between the fiber diameters measured by SEM for matrices of different organs. *, a significant difference between matrices, $p < 0.05$; —, no significant difference is found (one-way ANOVA with Holm-Sidak test for multiple comparisons)

	Liver	Lung	Spleen	Kidney	Ovary
Collagen	*	*	*	*	*
Ovary	—	—	*	—	
Kidney	—	*	*		
Spleen	*	*			
Lung	—				

Figure S5. Results of statistical comparison between the macroindentation results of different organs. *, a significant difference between matrices, $p < 0.05$; —, no significant difference is found (one-way ANOVA with Holm-Sidak test for multiple comparisons)

	Lung	Spleen	Kidney	Ovary
Liver	*	—	—	—
Ovary	*	—	—	
Kidney	—	—		
Spleen	—			

Figure S6. Results of statistical comparison between the nanoindentation results of different organs. *, a significant difference between matrices, $p < 0.05$; —, no significant difference is found (one-way ANOVA with Holm-Sidak test for multiple comparisons)

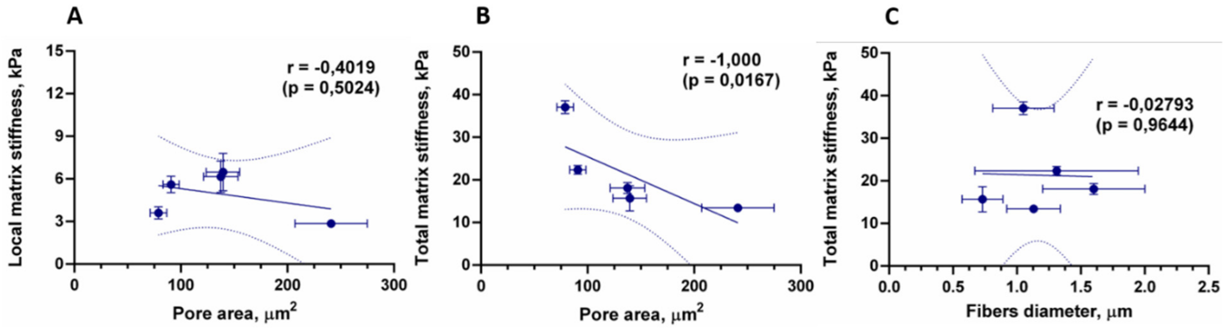

Figure S7. Analysis of the correlation between the matrix stiffness and pore area or fibers diameter

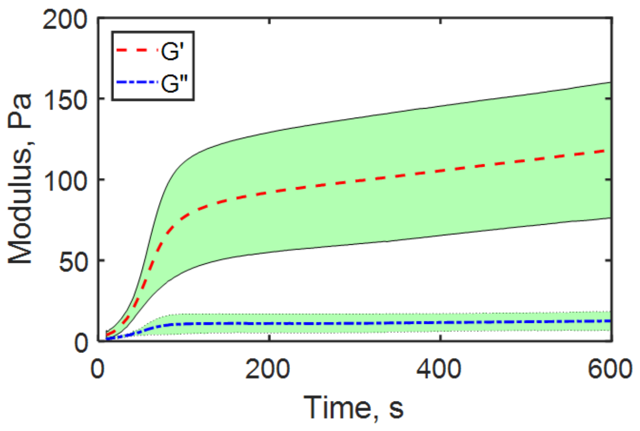

Figure S8. Shear rheology of collagen hydrogel measured using a Physica MCR 302 rheometer (Anton Paar, Austria) in oscillatory mode at room temperature in a chamber with high humidity; storage modulus, G′ (Pa), and loss modulus, G″ (Pa), were measured (n=3). This method has allowed the estimation of the collagen gel formation time. The substantial growth of the shear modulus was finished in 60-80 seconds. The storage and loss of shear moduli after 10 minutes were 119±63 Pa and 13±6 Pa, respectively. Storage modulus is an elastic component, loss modulus is a viscous component. The greater the shift towards storage modulus, the more elastic the object is; in the case of collagen gel, there are more cross-links

between the fibrils. Based on the Poisson's ratio of 0.5 for the collagen hydrogels, these values can be translated into Young's modulus of 360±190 Pa

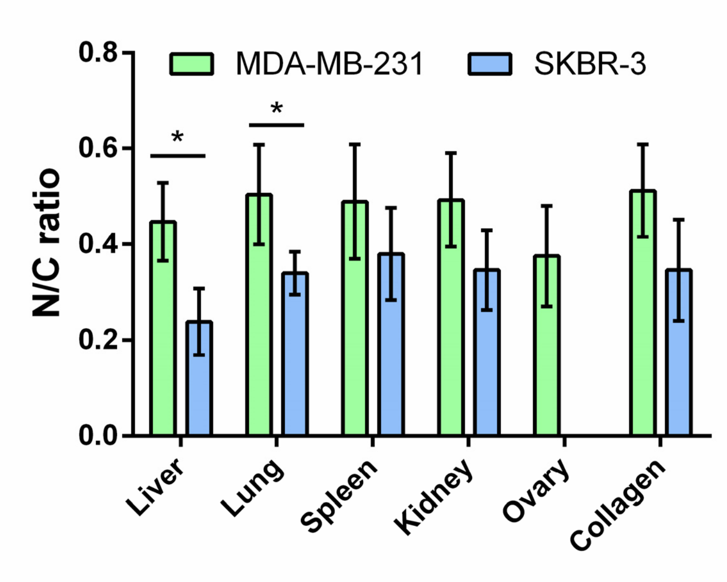

Figure S9. The nuclear-cytoplasmic ratio for MDA-MB-231 and SK-BR-3 cells in matrices of different organs. *, a significant difference between cell lines (Mann Whitney two-tailed test, $p < 0.05$).

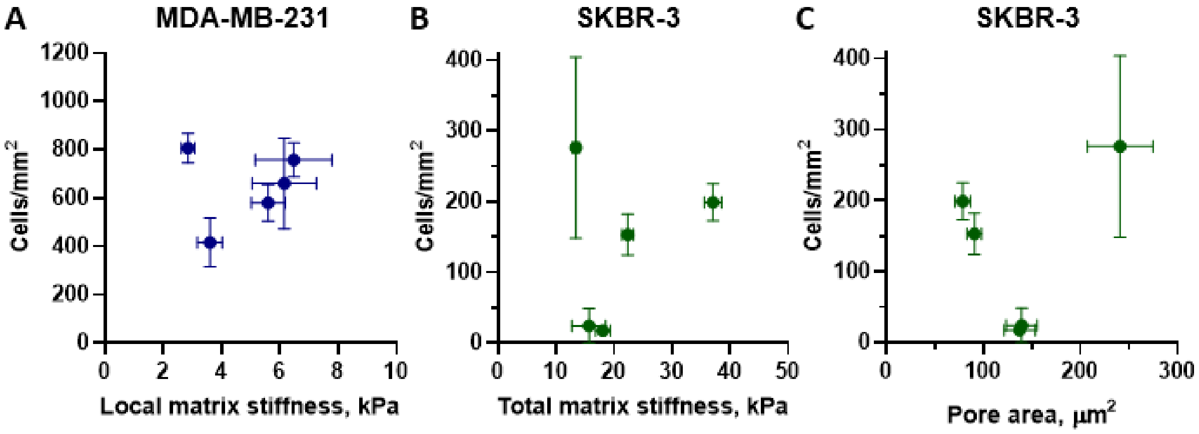

Figure S10. Analysis of the correlation between the matrix stiffness or pore area and the number of repopulating breast adenocarcinoma cells. (A) Local matrix stiffness vs. the number of MDA-MB-231 cells. (B) Total matrix stiffness vs. the number of SKBR-3 cells. (C) Pore area vs. the number of SKBR-3 cells.;
